# Auditory Cognitive Training Improves Brain Plasticity in Healthy Older Adults: Evidence From a Randomized Controlled Trial

**DOI:** 10.3389/fnagi.2022.826672

**Published:** 2022-03-31

**Authors:** Natasha Y. S. Kawata, Rui Nouchi, Kentaro Oba, Yutaka Matsuzaki, Ryuta Kawashima

**Affiliations:** ^1^Department of Functional Brain Imaging, Institute of Development, Aging and Cancer (IDAC), Tohoku University, Sendai, Japan; ^2^Department of Cognitive Health Science, Institute of Development, Aging and Cancer (IDAC), Tohoku University, Sendai, Japan; ^3^Smart Aging Research Center, Tohoku University, Sendai, Japan; ^4^Department of Human Brain Science, Institute of Development, Aging and Cancer (IDAC), Tohoku University, Sendai, Japan; ^5^Department of Developmental Cognitive Neuroscience, Institute of Development, Aging and Cancer (IDAC), Tohoku University, Sendai, Japan

**Keywords:** auditory-cognitive training, cognitive function, auditory ability, older adults, temporal pole

## Abstract

The number of older adults is increasing globally. Aging is associated with cognitive and sensory decline. Additionally, declined auditory performance and cognitive function affect the quality of life of older adults. Therefore, it is important to develop an intervention method to improve both auditory and cognitive performances. The current study aimed to investigate the beneficial effects of auditory and cognitive training on auditory ability and cognitive functions in healthy older adults. Fifty healthy older adults were randomly divided into four training groups—an auditory-cognitive training group (AC training; *n* = 13), an auditory training group (A training; *n* = 13), a cognitive training group (C training; *n* = 14), and an active control group (*n* = 12). During the training period, we reduced the sound intensity level in AC and A training groups and increase training task difficulty in AC, A, and C training groups based on participants’ performance. Cognitive function measures [digit-cancelation test (D-CAT); logical memory (LM); digit span (DS)], auditory measures [pure-tone audiometry (PTA)], and magnetic resonance imaging (MRI) scans were performed before and after the training periods. We found three key findings. First, the AC training group showed difference between other training groups (A, C, and active control training groups) in regional gray matter volume (rGMV) in the right dorsolateral prefrontal cortex, the left inferior temporal gyrus (L. ITG), the left superior frontal gyrus, the left orbitofrontal cortex, the right cerebellum (lobule 7 Crus 1). Second, the auditory training factor groups (ATFGs, the AC and A training groups) improved auditory measures and increased the rGMV and functional connectivity (FC) in the left temporal pole compared to the non-ATFGs (the C training group and active control group). Third, the cognitive training factor groups (CTFGs; the AC and C training groups) showed statistically significant improvement in cognitive performances in LM and D-CAT compared to the non-CTFGs (the A training group and active control group). Therefore, the auditory training factor and cognitive training factor would be useful in enhancing the quality of life of older adults. The current AC training study, the plasticity of the brain structure was observed after 4 weeks of training.

## Introduction

Aging is associated with cognitive and sensory decline (Deal et al., 2017). The incidence of age-related hearing loss (ARHL) has been increasing among older adults worldwide. Hearing loss has become a public health problem with the burgeoning aging population (WHO, 2011). ARHL is characterized by degeneration of the mechanotransducing inner and outer hair cells of the cochlea as well as the auditory nerve (neural presbycusis) (Schuknecht and Gacek, 1993; Ohlemiller, 2004). In addition to peripheral lesions, changes are likely to occur in the central auditory pathways, and these contribute to the development and progression of ARHL (Jayakody et al., 2018). ARHL is a multifactorial disorder with several underlying risk factors, such as age, environment, and lifestyle (Yamasoba et al., 2013). ARHL causes speech perception problems (Lin, 2011) and has been linked to consequent decline in cognitive function (Deal et al., 2017; Kawata et al., 2021), increased social isolation (Mick et al., 2014), reduced quality of life (Li et al., 2014), increased risk of depression (Li et al., 2014), and decline in ability to independently perform activities of daily living (Dalton et al., 2003). Epidemiological evidence across populations suggests that cognitive decline with ARHL is a risk factor for the development of dementia in older adults (Taljaard et al., 2016).

Speech comprehension involves the perceptual sensitivity of the peripheral nervous system and the language-specific cognitive abilities of the central nervous system (Sommers, 1997). Success in achieving listening goals may depend on the distribution of greater cognitive functions of the listeners and the quality of the signals (Pichora-Fuller et al., 2016). Therefore, it is important to consider the aspects of both hearing and cognitive functions. Multiple factors (such as hearing loss and decline in cognitive function) contribute to speech recognition difficulties (Wayne and Johnsrude, 2015; Kawata et al., 2020). When a speech signal is poorly processed, it is transmitted from the ear to the brain, and greater cognitive resources may be required to interpret the meaning of the sound than would be with a properly processed sound (Schneider et al., 2010). Thus, there is no guarantee that increasing cognitive energy will solve hearing problems. Therefore, success in achieving auditory goals may depend on the considerable cognitive energy expenditure that is required when the quality of the signal available to the listener is suboptimal. Therefore, it would be important to improve cognitive function as well as auditory sensory function. One approach is using a combination of auditory and cognitive training (AC training). Recently, an AC training has been proposed (Yusof et al., 2019). Yusof used AC training to improve speech recognition, auditory processing, and cognitive abilities in older adults with normal cognitive and mild cognitive impairment (Yusof et al., 2019). Yusof used five adaptive training tasks (word-in-noise, sentence-in-noise, word span, word order, and word position). After 8 weeks’ of AC training, improvements in general cognitive functions measured by Montreal Cognitive Assessment and auditory processing ability measured by a dichotic digits test was observed in older adults with normal cognition and neurocognitive impairment compared to that of a control group.

A previous study reported that AC training had positive effects on cognitive and auditory functions (Yusof et al., 2019). However, some limitations were noted. First, they did not directly measure auditory abilities using objective auditory assessment measures, such as the pure-tone audiometry (PTA) threshold. Second, they did not use a suitable active control group. In the previous study, the control group participants were asked to watch documentary programs on history and literature using the same device used for AC training (Yusof et al., 2019). This ensured that the control and training groups matched in terms of training duration and the auditory stimuli received. However, it is unclear which components of the auditory and cognitive aspects are important for improving cognitive and auditory performance. Third, the previous study used different signal-to-noise-ratio to modify the difficulty of the task (Yusof et al., 2019). Although it is important to listen to the words and sentences with noises (Pichora-Fuller et al., 1995), older adults usually show difficulty with low sound intensity level [decibel (dB)] (Slade et al., 2020). Fourth, previous study did not investigate whether AC training affect neural systems. It is still unclear that AC training would have positive effects on brain functions and brain structures.

The present study was designed to evaluate the beneficial effects of AC training on hearing ability and cognitive and brain functions in healthy older adults. We conducted a single-blinded randomized controlled trial using an AC training group, an auditory (A) training group, a cognitive (C) training group, and an active control group. Considering the abovementioned shortcomings of the previous studies, we used objective auditory measures (PTA) to assess auditory abilities. Moreover, to resolve the issue with the active control group, we used an active control group that eliminated the need for change in cognitive or auditory training difficulty. In this study, we controlled the sound intensity level to manipulate the auditory factor during training. By controlling the sound intensity level, we were able to control auditory training factor in the AC and A training groups. Furthermore, we conducted magnetic resonance imaging (MRI) before and after training to investigate changes in brain plasticity after training. In the field of cognitive aging, the application of structural and functional connectivity of the brain is particularly important because these brain changes usually precede behavioral changes (Farras-Permanyer et al., 2019). In addition, previous studies demonstrated that cognitive training led to change brain structures (gray matter volume) and functional connectivity after interventions (Chaddock-Heyman et al., 2021; Yin et al., 2021). Based on the previous finding, we investigated brain plasticity using MRI with change in the regional gray matter volume and the resting-state functional connectivity.

The present study conducted a training period of 4 weeks. Previous studies of auditory training performed auditory training for 4 weeks (Karawani et al., 2016). Significant improvements were observed in all training conditions in both the ARHL and normal hearing groups. Improvements in assessments of cognitive function after 4 weeks of training have been reported in a study of cognitive training in the elderly (Biel et al., 2020). Moreover, the previous AC training study conducted training for 8 weeks (Yusof et al., 2019). The participants were evaluated after 4 and 8 weeks of training. Both the normal hearing and hearing loss groups were shown to have improved auditory processing and cognitive function. In a cognitive training study, the plasticity of the brain structure was observed after 4 weeks of training (Biel et al., 2020). For this reason, the present study conducted training for 4 weeks.

This is the first study to use AC training with a lower intensity level than the subjective comfortable listening level to control the difficulty in auditory training factor. To evaluate the beneficial effect of AC training on auditory ability and cognitive and brain functions in healthy older adults, we used PTA to measure auditory performance, cognitive measures (D-CAT, LM, DS), and brain structure and functional connectivity (FC).

We proposed the following three hypotheses regarding the behavioral change and the brain plasticity from each training group. First, we hypothesized that AC training would show a superior beneficial effect on cognitive and auditory performances than would the other training groups because cognitive (tasks difficulty during training) and auditory (sound intensity level control) factors were changed based on participants’ performance. For the brain plasticity, we also expected that AC training would alter regional gray matter volume (rGMV) and FC related to cognitive and auditory processes. For example, we expected brain plasticity in the bilateral temporal cortex (speech perception) (Peelle, 2019), the temporal pole (TP), and the precuneus, that are related to auditory processes (high listening effort) (Olson et al., 2007; Rosemann and Thiel, 2019). Moreover, we expected brain plasticity in the dorsal attention network (DAN) (Sanchez-Perez et al., 2019), prefrontal regions (Nissim et al., 2017), and medial temporal lobe (MTL), that are related to high cognitive processes (Tsukiura et al., 2002). Second, we assumed that the auditory training factor groups (ATFGs; the AC and A training groups) would show improvement in the auditory performance and the brain plasticity in the abovementioned auditory process-related brain regions. Third, we hypothesized that the cognitive training factor groups (CTFGs; the AC and C training groups) would show improvements in the cognitive performance and the brain plasticity in the abovementioned cognitive process-related brain regions because previous cognitive training studies have reported the beneficial effects of cognitive training on several cognitions in the healthy older adults (Nouchi et al., 2016b; Alnajjar et al., 2019).

## Experimental Methods

### Ethics Statement

This study was registered in the UMIN Clinical Trial Registry (UMIN000042271). It was conducted between August 2019 and December 2019 in Sendai City, Miyagi Prefecture, Japan.

**FIGURE 1:**
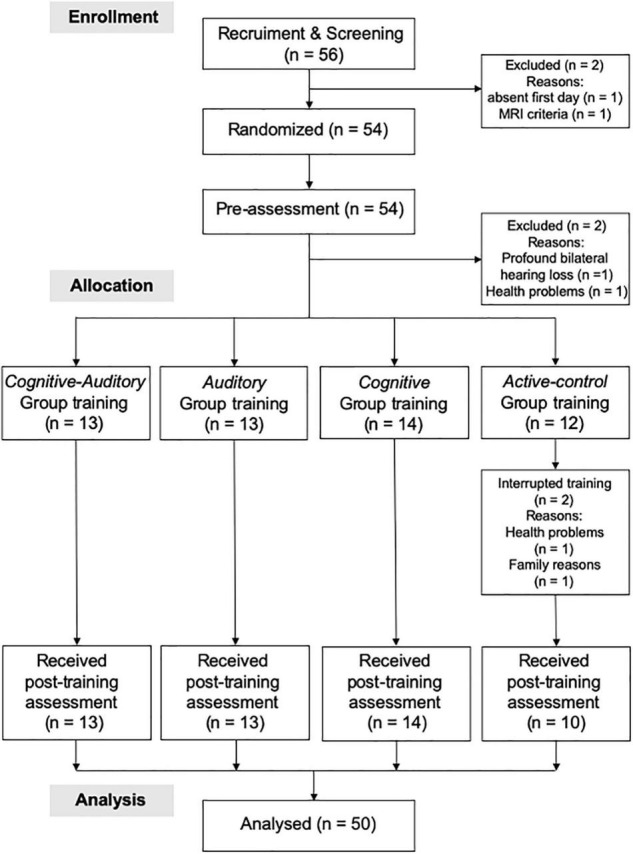
CONSORT flowchart.

Participants were informed that the study was designed to investigate the effects of four training programs. The researchers who were not involved in generating the randomization sequence enrolled eligible participants and conducted pre-assessments. These participants were then randomly assigned to receive combination training (AC training group), single training (A training group or C training group), and no training (active control group). The researchers (who were not involved in generating the randomization sequence) enrolled and allocated participants to either the A (C training group), B (A training group), C (AC training group) or D (active control group) letters. The random allocation sequence was generated using an online computer program.^1^ All participants engaged in their assigned training during in-person visits for 4 weeks. After training, the participants completed the post-training outcome assessments (Figure 1).

### Participants

Fifty-six participants [13 men and 43 women; mean age = 68.07 years (standard deviation, *SD* = 4.14)] were recruited from the general population through advertisements in a local town paper and local newspapers (Kahoku Weekly). Interested participants were screened using a semi-structured telephone interview (10 questions) that approximately 10 min. The 10 questions pertaining to the inclusion and exclusion criteria were related to (1) age, (2) sex, (3) previous experience in intervention studies, (4) native language, (5) handedness, (6) subjective memory function, (7) history of medication use and disease (including hearing-related problems), (8) blood pressure, (9) history of diabetes, and (10) ability to complete training schedule. The participants were then invited to visit Tohoku University. We collected written informed consent from 55 participants (one participant did not visit the institution on the first day). Subsequently, all participants were subjected to a detailed auditory assessment [including assessment of PTA threshold and speech reception threshold (SRT)], cognitive function tests [such as the Mini-Mental State Examination (MMSE), LM, DS, and D-CAT], and MRI. None of the participants were excluded based on MMSE scores. However, two participants declined to participate before they were randomized into groups. One participant was excluded based on the auditory assessment (profound unilateral hearing loss), one participant was excluded because the MRI examination criteria were not met, and two participants declined to participate after initiating training (Figure 1).

#### Overview of Auditory Measures

To investigate the effect of auditory sensitivity after performed auditory training, we performed the PTA measurement, before and after the training. All participants underwent auditory assessment before starting the training schedule and after completing the eight training sessions at Tohoku University in a soundproof room. The PTA air conduction thresholds were measured using an audiometer (AA-76, RION, Tokyo, Japan) and standard headphones (AD-06B). The audiometer was calibrated in dB hearing level according to standards of the International Organization for Standardization (ISO/TC, 1996) and the American National Standard Institute (2019). Before PTA, all participants underwent an otoscopic examination to exclude occluded ear canals or other irregularities (i.e., no tympanic membrane abnormalities were observed). Each ear was assessed. Details of these measures are described in Supplementary Material.

#### Overview of Cognitive Function Measures

To measure the effects of cognitive training on each training group, participants performed the follow cognitive measures. Cognitive function was divided into four categories: general cognition, episodic memory, working memory, and attention. Global cognitive status was measured using the MMSE (Folstein et al., 1975). Episodic memory was measured using LM (Wechsler, 1987). Working memory was measured using DS (Wechsler, 1997). Attention was measured using D-CAT (Hatta et al., 2001). Details of these measures are described in Supplementary Material.

#### Magnetic Resonance Imaging Data Acquisition and Imaging Parameter

To acquire MRI data, we used a 3.0 Tesla Philips Achieva MRI scanner (Philips, Amsterdam, The Netherlands) with an eight-channel head coil at the Institute of Development, Aging and Cancer, Tohoku University. Fifty participants performed MRI before and after assessment. They were instructed to avoid moving their head. High-resolution T1-weighted structural images [240 × 240 matrix, time repetition (TR) = 6.6 ms, time echo (TE) = 3 ms, field of view (FOV) = 24 cm, slices = 162, and slice thickness = 1 mm] were collected using a magnetization-prepared rapid gradient-echo sequence. The quality of all imaging data was checked visually. The total scan time was 8 min. For the resting-state parameter, we used 34-transaxial gradient-echo images (64 × 64 matrix, TR = 2,000 ms, TE = 30 ms, flip angle = 70°, FOV = 24 cm, and slice thickness = 3.75 mm) covering the entire brain and acquired using an echo-planar sequence. For this scan, 160 functional volumes were obtained, while the participants were resting. The total scan time was 6 min. we utilized the same parameters as those used in a previous laboratory study (Takeuchi et al., 2012). During resting-state scanning, the participants were instructed to keep their eyes closed, stay as motionless as possible, not fall asleep, and avoid thinking about anything in particular.

#### Inclusion and Exclusion Criteria

Based on the previous intervention studies for older adults (Nouchi et al., 2019, 2021), the following participants were included: those who self-reported being right-handed, those who were native Japanese speakers; those who were unconcerned about their memory function; those who were not taking medications that interfered with cognitive function (such as benzodiazepines, antidepressants, and other central nervous system agents); those who did not have a history of diseases that affect the central nervous system, including thyroid disease, multiple sclerosis, Parkinson’s disease, stroke, severe hypertension (systolic blood pressure > 180 mmHg and diastolic blood pressure > 110 mmHg), and diabetes; and those who were > 60 years old. Participants who had participated in other cognitive or auditory intervention studies were excluded. Participants with an MMSE score of < 26 (Folstein et al., 1975) or those with moderate-to-profound hearing loss were also excluded.

### Training Materials

#### Four Training Groups

**FIGURE 2:**
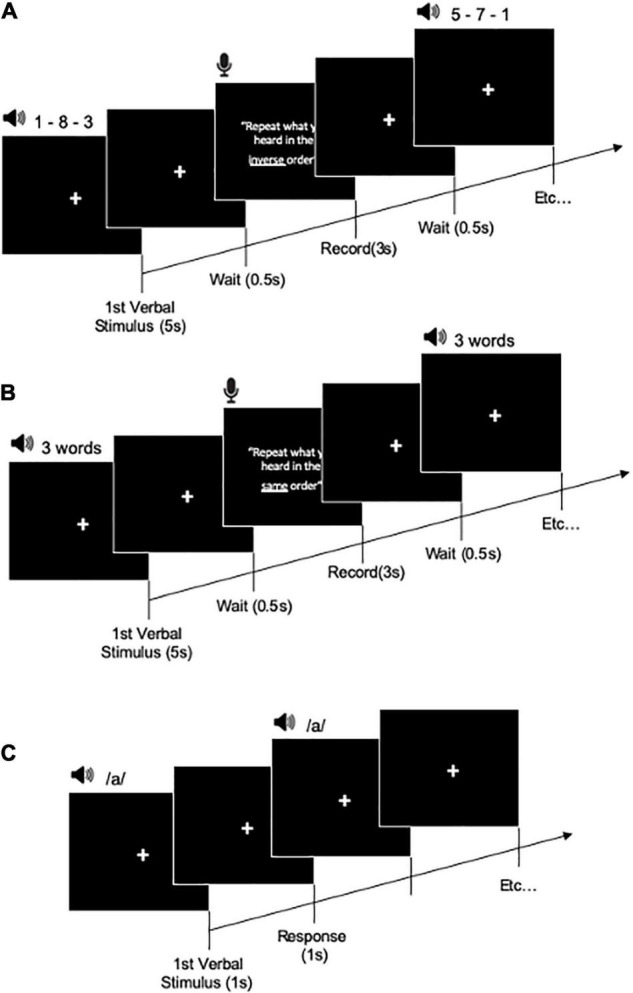
Training task procedure for working memory training task **(A)**, short-term memory training task **(B)**, and attention training task **(C)**.

We set four training groups (AC training, A training, C training, and active control groups) (Table 1). All training groups performed three cognitive training tasks (short-term memory, working memory, and attention training tasks) with intensity level controlled audio stimuli (Figure 2). The short-term, working memory, and attention training tasks were developed based on our previous cognitive training studies (Nouchi et al., 2016a, 2019, 2020a, 2021, 2022; Takeuchi et al., 2020). Each cognitive training task had 4 task difficulty levels as a cognitive training factor [from level 1 (easy) to level 4 (difficult)]. Sound intensity level of auditory stimulus had 4 levels as an auditory training factor [from level 1 (easy) to level 4 (difficulty)].

**TABLE 1:** Based on the three cognitive task training (cognitive training factor) and audio stimuli intensity level control (auditory training factor), we set four training groups [auditory-cognitive training (AC), auditory training (A), cognitive training (C), active control groups].

Training group	Auditory training factor	Cognitive training factor
AC training	+	+
A training	+	–
C training	–	+
Active control group	–	–

*The “+” symbol means that it contains variations in level difficulty. The “–” symbol means that it does not contain ant variation in the degree of level difficulty.*

The AC training group underwent a combination of cognitive and auditory training. In this group, the levels of the cognitive and auditory training factors were changed at the same time from level 1 to level 4 depending on the participants’ performance.

In the A training group, the auditory training factor varied from level 1 to level 4 depending on the participants’ performance. However, the cognitive training factor was not changed. The participants completed the three cognitive training tasks at level 1 difficulty of the cognitive training factor.

In the C training group, the cognitive training factor varied from level 1 to level 4 depending on the participants’ performance. However, the auditory training factor did not change. The participants completed the three cognitive training tasks at level 1 of the auditory training factor.

In the active control group, there were no variations in the cognitive or auditory training factors. The participants completed the three cognitive training tasks at level 1 of cognitive and auditory training factors in all the training sessions.

#### Criteria of Level Change of Cognitive and Auditory Training Factors

We checked the participants’ performance after each session. Levels of cognitive and auditory training factors were changed in every training session. The training level was increased when performance was > 70%; it was maintained when the performance on the training tasks was 50–70% and was decreased by one level when the performance was < 50%.

#### Manipulation of Sound Intensity Level of Auditory Stimulus

All three cognitive training tasks used auditory stimulus. Stimuli were presented in both ears. In the first session, all participants completed the three cognitive training tasks at level 1 of the auditory training factor. The level 1 of the auditory training factor differed among the participants because the level 1 of auditory factor was set based on the SRT at baseline.

For, the C training and active control groups the auditory training factor levels were not changed throughout the training sessions. They completed each cognitive training task at the level 1 of the auditory training factor. In the AC and A training groups, the sound intensity level of auditory stimuli for each cognitive training task was changed based on the cognitive training performance.

In this study, level 1 sound intensity level was set at SRT + 3 dB to SRT—3 dB. Level 2 sound intensity level was set at SRT + 0 dB to SRT—6 dB. Level 3 sound intensity level was set at SRT—3 dB to SRT—9 dB. Level 4 sound intensity level was set at SRT—6 dB to SRT—12 dB.

### Details of Each Training Task

The subjects performed the tasks in the following order: short-term memory task, working memory task, and attention task. There were no criteria that decided the order. However, all subjects performed the same order. In addition, we selected the short-term/episodic memory training using words stimulus and working memory training using numbers stimulus based on previous cognitive training studies such as working memory training (Takeuchi et al., 2013) and short-term/episodic memory training (Ball et al., 2002). Also, to avoid a confusion of both training procedure, we used different types of stimuli between working memory training and short-term/episodic memory training. We expected that participants can easily understand the differences of training task procedure and perform trainings without confuse.

#### Short-Term and Episodic Memory Training Task

Short-term and episodic memory training task was performed using a word recall task. After listening to each list of words, the participants were asked to recall the words they could remember from the list in the same order. The recall of each trial was recorded. If all words were repeated in the correct order in a trial, then a score of 1 was given; if the response was incorrect in any way, then the trial was assigned a score of 0. In one training session, the participants performed all the tasks three times for 5 min. Level 1 was composed of three words. Level 2 comprised four words. Level 3 included five words, and level 4 was composed of six words.

#### Working Memory Training Task

Working memory training task included the most commonly used listening span training. Participants listened (via headphones) to numbers and then recalled all numbers in reverse order. The recall was made orally and recorded. There was no time limit for responding. The subsequent trial started only when the participants pressed a button. If all digits in atrial were recalled in the correct order, then the trial was given a score of 1; if the response was incorrect in any way, then the trial was assigned a score of 0. In one training session, the participants performed all tasks three times for each task for 5 min. Level 1 of cognitive training was composed of three numbers. Level 2 was comprised four numbers. Level 3 was composed of five numbers, and level 4 six numbers.

#### Attention Training Task

In the attention training task (go/no-go attention task), the participants were presented one spoken vowel (i.e.,/a/) through headphones. The participants were instructed to press a “red button” key as quickly as possible each time a specific target vowel was presented. The response time (RT) and number of correct responses were recorded. In one training session, the participants performed the task three times for 5 min. Level 1 comprised one vowel and one target. Level 2 was composed of two vowels and one target. Level 3 involved three vowels and two targets. Level 4 was consisted of four vowels and two or three targets. The attention training task is similar task used in auditory attention training studies (Tallus et al., 2015).

#### Training Session Schedule

Each training session involved approximately 1 h of training per day was conducted 2 days per week for 4 weeks, resulting in a total of 8 session in predetermined order. The examiner and participants contacted each other by telephone in case of health problems (rescheduling the training date) and delays on the training session day. On the training day, it was possible to train two participants simultaneously for 1 h (two soundproof rooms were available).

#### Sample Size

No previous studies are using combined auditory and cognitive training on hearing ability, cognitive function, and brain plasticity in healthy older adults. Therefore, we did not conduct the sample size estimation for this study with accuracy. However, similar studies using cognitive training have been reported the improvements of cognitive functions after training. For example, the combined auditory and cognitive training with 16 participants led to the improvement in cognitive function (Yusof et al., 2019). Moreover, 4-week cognitive training study using 14 participants also reported cognitive improvements in processing speed and executive functions (Nouchi et al., 2012). For this reason, the present study was set from 14 to 16 participants in each group.

### Analysis

#### Behavioral Data Analysis

Table 2 presents the baseline characteristics of the participants. We calculated the changes in scores (post-assessment score minus pre-assessment score) for all cognitive function tests and auditory assessments. Cognitive function measures and auditory measures were dependent variables. We used a two (the auditory training factor: with/without) by two (the cognitive training factor: with/without) factorial analysis covariance (ANCOVA) with permutation tests to investigate significant group differences in each cognitive function measure and auditory measure. All analyses were performed using the “aovp” function of the “lmPerm” package for changes in scores associated with each cognitive measure and auditory measure. We used the permutation ANCOVA test because it is suitable for small sample analysis and is freely distributed. Therefore, the permutation ANCOVA test is suitable and sufficiently powered for present study (Kulason et al., 2018; Nouchi et al., 2020b, 2022). The changes in scores in each group (AC training group, A training group, C training group, and active control group) were the dependent variables. All pre-assessment scores of the dependent variables, sex, age, and MMSE were used as covariates to adjust for background characteristics and exclude the possibility of any pre-existing difference in measures between the groups affecting the result. Fifty randomly allocated participants were included in the analyses. The level of significance was set at *p* < 0.05. The PTA threshold was the primary outcome. We applied the Bonferroni–Holm procedure (Holm, 1979) separately for cognitive measures (LM, D-CAT, and DS findings) and auditory measures (PTA threshold). All analyses are performed using the R software (RStudio Team, 2019) (R Core Development Team, Toulouse, France).

**TABLE 2:** characteristics of participants in the AC training group, A training group, C training group, and active control group.

	AC-training group			A-training group			C-training group			Active control group			Max value
	Mean	*SD*	95% CI	Mean	*SD*	95% CI	Mean	*SD*	95% CI	Mean	SD	95% CI	
**Global cognitive status**													
MMSE (score)	28.77	1.05	[28.10, 29.42]	29.31	0.82	[28.79, 29.82]	29.36	0.81	[28.87, 29.84]	6.8	1.6	[26.59, 29.00]	30
**Working memory**													
DS (score)	7.07	2.26	[5.26, 8.30]	6.69	1.97	[5.44, 7.93]	6.78	2.54	[5.64, 8.50]	5.4	1.68	[4.12, 6.67]	16
**Episodic memory**													
LM (score)	10.46	3.02	[8.03, 13.11]	9.53	3.41	[7.39, 11.68]	10.57	4.36	[8.55, 12.36]	8.4	4.36	[5.10, 11.69]	25
**Attention**													
D-CAT (score)	172.9	21.44	[148.28, 190.64]	168.6	34.98	[146.60, 190.62]	174.8	44.22	[159.43, 186.41]	164.1	44.08	[130.85, 197.340]	200
**Pure-tone audiometry**													
PTA (dB)	16.44	7.58	[-2.80, 1.27]	16.54	6.28	[-2.08, 1.27]	21.43	10.71	[-2.41, 1.12]	23.88	7.86	[-2.46, 1.12]	90*

*Maximum output limits of the audiometer. AC, Auditory-cognitive training; A, auditory training; C, cognitive training; MMSE, Mini-Mental State Examination; DS, digit span; LM, logical memory; D-CAT, digit cancelation; PTA, pure-tone audiometry).

Additionally, participants in the active control group performed the baseline (level 1) in all sessions. In the behavioral analysis, we excluded the effect of active control on behavioral measures. Therefore, we excluded the possibility that the effect of active control had an impact on any outcome.

#### Image Preprocessing of Structural Brain Image

As a first step, we reviewed and converted all pre- and post-Digital Imaging and Communication in Medicine scans into the Neuroimaging Informatics Technology Initiative format using MRICRON software before running the analysis. All imaging data were analyzed using Statistical Parametric Mapping 12 (SPM12; Wellcome Department of Cognitive Neurology; London, United Kingdom) implemented in MATLAB (MathWorks Inc., Natick, MA, United States). Briefly, SPM12 and Computational Anatomy Toolbox 12 (CAT12)^2^ were used to create an asymmetric diffeomorphic anatomical registration through exponentiated Lie (DARTEL) algebra template from the original and flipped gray matter and white matter segments. T1-weighted structural images of each participant (pre- and post-imaging data) were segmented and normalized to the Montreal Neurological Institute (MNI) space using CAT12 to generate images with 1.5 × 1.5 × 1.5 mm^3^ voxel size diffeomorphic anatomical registration through the DARTEL registration process. Moreover, we performed volume change correction (modulation). We used a SPM12 image calculator (ImCalc) to calculate the post-imaging value minus pre-imaging value for all participants. The required mask expression was (i2—i1).*(i2 > 0.1).*(i1 > 0.1). The mask expression was used to restrict the statistical analysis to regions of the brain expected to contain true signals. Subsequently, the generated rGMV image was smoothed using a Gaussian kernel of 8-mm full width at half maximum (FWHM).

#### Brain Structural Statistical Analysis

Full factorial model analysis was performed using SPM12 and CAT12. This approach was used to analyze the superior effects of AC training compared to other training groups, the effect of the auditory training factor (with/without), and the cognitive training factor (with/without). The main effects of both the factors and group comparisons were used as contrasts of interest (cognitive training factor main effect: AC + C > A + active control; auditory training factor main effect: AC + A > C + active control; the superior effects of AC training compared to other training groups: AC > A + C + active control). The model included two levels of each factor (cognitive and auditory training factors), age, sex, and total intracranial volume as covariates. Additionally, in the analysis of the superior effects of AC training compared to other training groups, we included the mask images (AC > C, AC > A, and AC > active control, a threshold of *p* < 0.05, uncorrected). Furthermore, we analyzed the relationship between behavioral changes scores (auditory and cognitive measures) and GMV. The covariates were mean centered, and we used threshold-free cluster enhancement (TFCE) with randomized (5.000 permutations) non-parametric testing using the TFCE toolbox.^3^ We applied a cluster-level FWE-corrected at *p* < 0.05 (Takeuchi et al., 2012).

#### Preprocessing and Analysis of Resting-State Functional Connectivity

Resting-state FC preprocessing and analysis were performed using a standard pipeline in the CONN toolbox (Whitfield-Gabrieli and Nieto-Castanon, 2012), implemented in MATLAB. Preprocessing included realignment, direct segmentation, normalization to the MNI space (2 mm^3^), outlier detection (artifact detection tool based identification of outlier scans for scrubbing; motion correction = 0.9 mm; global-signal *z*-value threshold = 5),^4^ and smoothing (FWHM = 8 mm). The realignment and scrubbing parameters and the BOLD signal from the WM and cerebrospinal fluid were regressed using a general linear model. Data were band-pass filtered at 0.008–0.09 Hz to reduce the effects of low-frequency drifts and high-frequency noise. First-level analyses included the calculation of individual whole-brain seed-to-voxel FC maps (Takeuchi et al., 2017).

#### Resting-State Functional Connectivity of Analysis

For second-level analysis, in the group-level comparisons, seed-based FC maps were used to analyze the superior effects of AC training compared to other training groups (AC > A + C + active control), main effect of the auditory training factor, and main effect of the cognitive training factor. We included the mask expression (AC > C, AC > A, and AC > active control, a threshold of *p* < 0.05, uncorrected). The brain seed regions were selected with reference to the results obtained in the brain structure analysis. Furthermore, we analyzed the relationship between behavioral changes scores (auditory and cognitive measures) and functional connectivity. We used TFCE with randomized (5.000 permutations) non-parametric testing using the TFCE toolbox (see text footnote 3). The clusters were threshold at an FWE corrected at *p* < 0.05 using a cluster-forming threshold of *p* < 0.001, which was uncorrected.

## Results

### Behavioral Data

**FIGURE 3:**
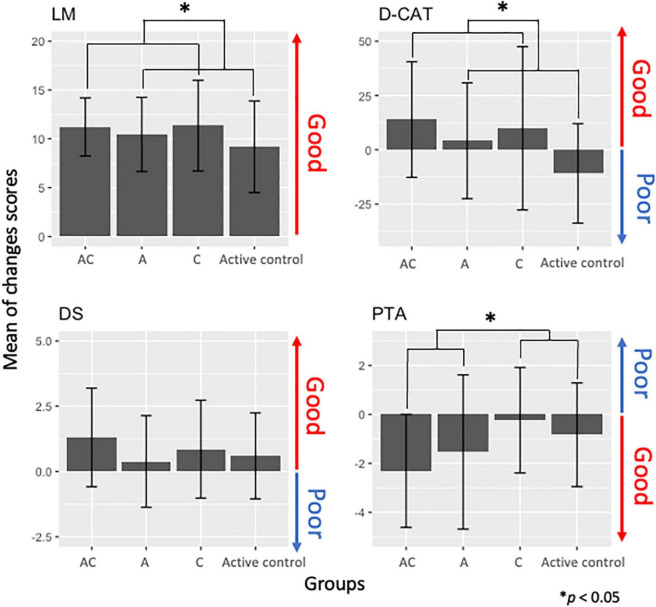
Change scores in cognitive function measures [logical memory (LM), digit cancelation (D-CAT), digit span (DS)] and auditory [pure-tone auditory (PTA)] in each training group [auditory-cognitive training (AC), auditory training (A), cognitive training (C)].

All participants had normal cognitive function, as indicated by MMSE scores [mean = 28.88, standard deviation (SD) = 1.22], and a normal to mild PTA threshold according to the Japan Audiological Society (mean = 19.23, *SD* = 2.95). Cognitive and auditory assessment scores before and after training in all groups are presented in Figure 3.

First, to investigate whether a group difference existed at the baseline (before training), we performed a two (the auditory training factor: with/without) by two (the cognitive training factor: with/without) ANCOVA with permutation tests for the baseline data. We did not find statistically significant interaction between the auditory and cognitive training factors {LM [*F*_(1, 44)_ = 0.49, *p* = 0.63, adjusted *p* = 0.85], D-CAT [*F*_(1, 44)_ = 0.17, *p* = 0.64, adjusted *p* = 0.85], DS [*F*_(1, 44)_ = 0.65, *p* = 0.51, adjusted *p* = 0.85], PTA [*F*_(1, 44)_ = 3.25, *p* = 0.08, adjusted *p* = 0.48]}. We did not find any significant effect of the auditory training factor {LM [*F*_(1, 44)_ = 0.02, *p* = 0.96, adjusted *p* = 0.96], D-CAT [*F*_(1, 44)_ = 0.04, *p* = 0.60, adjusted *p* = 0.48], DS [*F*_(1, 44)_ = 0.96, *p* = 0.23, adjusted *p* = 0.58], PTA [*F*_(1, 44)_ = 1.50, *p* = 0.17, adjusted *p* = 0.58]}, and the main effects of the cognitive training factor {LM [*F*_(1, 44)_ = 0.91, *p* = 0.26, adjusted *p* = 0.58], D-CAT [*F*_(1, 44)_ = 1.12, *p* = 0.9, adjusted *p* = 0.96], DS [*F*_(1, 44)_ = 1.37, *p* = 0.29, adjusted *p* = 0.58], PTA [*F*_(1, 44)_ = 0.021, *p* = 0.96, adjusted *p* = 0.96]}. The results indicated that the cognitive functions and auditory performance at baseline did not differ among the groups.

Second, we investigated effects of the interventions on cognitive function and auditory performance using the ANCOVA for changes in scores. We did not find statistically significant beneficial effects of AC training on PTA thresholds [*F*_(1, 43)_ = 0.72, *p* = 0.27, adjusted *p* = 0.40], LM [*F*_(1, 42)_ = 0.19, *p* = 0.38, adjusted *p* = 0.49], D-CAT scores [*F*_(1, 42)_ = 2.30, *p* = 0.06, adjusted *p* = 0.17], and DS [*F*_(1, 42)_ = 0.28, *p* = 0.92, adjusted *p* = 1.00] compared to other training groups. However, we found statistically significant main effects in the factor groups. In terms of cognitive functions, the CTFGs (the AC and C training groups) had improvements in LM [*F*_(1, 42)_ = 5.15, *p* = 0.009, adjusted *p* = 0.04] and D-CAT scores [*F*_(1, 42)_ = 7.2, *p* = 0.006, adjusted *p* = 0.04] compared to the non-CTFGs (Figure 3). Moreover, the ATFGs (the AC and A training groups) had improved auditory performance [*F*_(1, 42)_ = 3.12, *p* = 0.02, adjusted *p* = 0.06] compared to the non-ATFGs (Figure 3).

### Brain Structural Results

**FIGURE 4:**
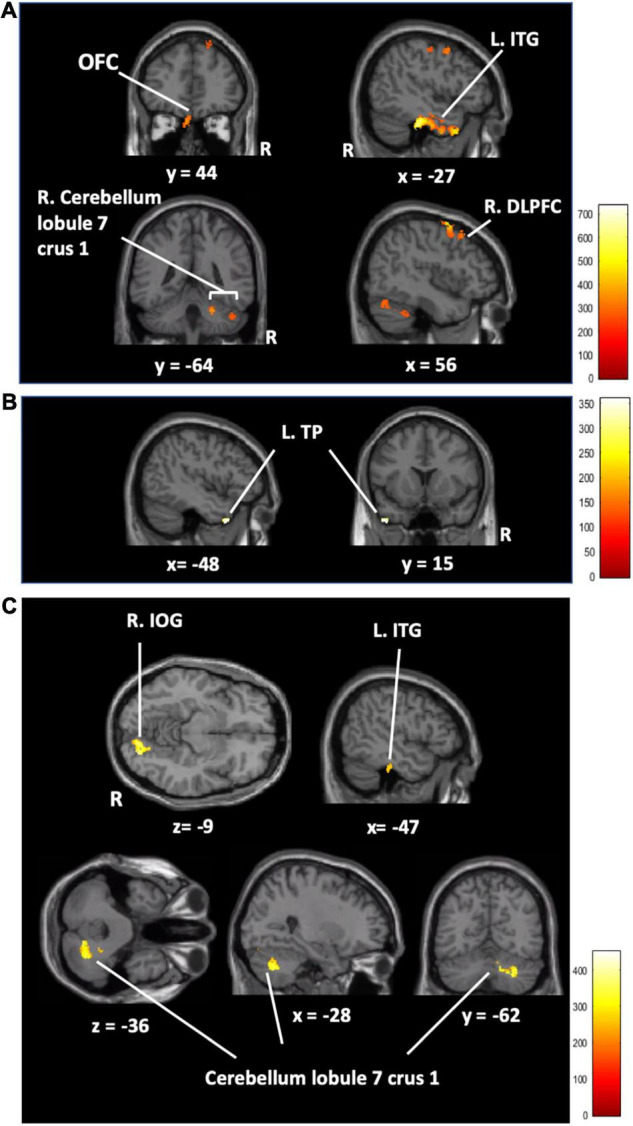
**(A)** The regional gray matter volume results in the AC training group compared to that in the other training groups (AC > A + C + active control). The regional gray matter volume results of the auditory training factor main effect **(B)** and cognitive training factor main effect **(C)**. [Orbitofrontal cortex (OFC), left inferior temporal gyrus (L.ITG), right superior frontal gyrus (R.SFG), left temporal pole (L.TP), right inferior occipital gyrus (R.IOG), auditory-cognitive training (AC), auditory training (A), cognitive training (C)] FWE corrected at *p* < 0.05 based on 5,000 permutations. The color represents the strength of the TFCE values.

Only the AC training group showed changes between other training groups (A, C, and active control training groups) in rGMV in the right dorsolateral prefrontal cortex (R. DLPFC), the left inferior temporal gyrus (L. ITG), the left superior frontal gyrus (L. SFG), the left orbitofrontal cortex (L. OFC), and the right cerebellum (lobe 7 Crus 1) (FWE corrected at *p* < 0.05, Figure 4 and Table 3). In addition, the ATFGs showed changes in the cluster located in the left temporal pole (L. TP) compared to the non-ATFGs (FWE corrected at *p* < 0.05, Figure 4 and Table 3). Differences were observed in the clusters located in the right inferior occipital gyrus (R. IOG), right cerebellum (lobule 7 Crus 1) and R. ITG between the CTFGs and non-CTFGs (FWE corrected at *p* < 0.05, Figure 4 and Table 3). In addition, we analyzed the relationships between behavior (cognitive and auditory) changes and brain structural changes. However, any changes of auditory and cognitive measures did not significantly correlate with brain structure changes.

**TABLE 3:** Brain regional gray matter volume with a significant cluster in main effects analysis and group comparison analysis.

			Peak MNI coordinates		
Anatomical	Cluster size	Corrected *p*-value (FWE)	x	y	z
location	(mm^3^)				
**AC > A + C + active control**					
R. DLPFC	747	0.005	56	–5	51
L. ITG	2,554	0.001	–48	–27	–29
L. SFG	682	0.025	–48	6	53
L. OFC	184	0.017	–5	44	–32
R. Cerebellum Lobule 7 Crus 1	1,610	0.002	12	–87	–20
**The main effect of the auditory factor**					
L. TP	81	0.021	–48	15	–42
**The main effect of the cognitive factor**					
R. IOG	893	0.005	14	–81	–14
R. Cerebellum R. Lobule 7 Crus 1	35	0.036	38	–78	–23
ITG	71	0.032	–47	-29	–32

*R. DLPFC, Right dorsolateral prefrontal cortex; L.ITG, left inferior temporal gyrus; L. SFG, left superior frontal gyrus; L. OFC, left orbitofrontal cortex; 7 crus1, cerebellum lobule; L. TP, left temporal pole; R. IOG, right inferior occipital gyrus; AC, auditory-cognitive training; A, auditory training; C, cognitive training) FWE corrected at p < 0.05.*

### Brain Functional Connectivity Results

**FIGURE 5:**
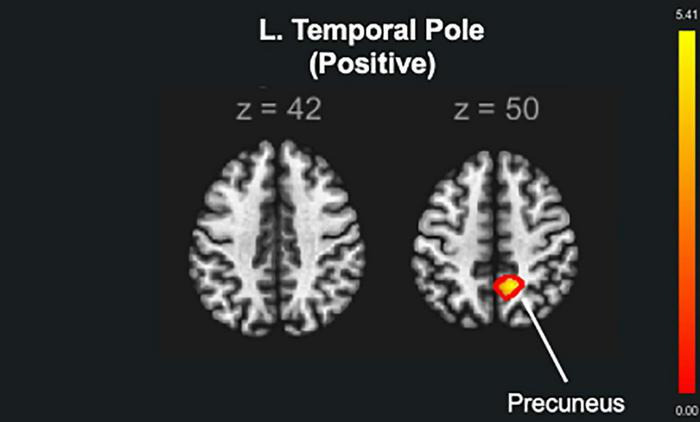
The functional brain connectivity of the auditory training factor groups (ATFGs) compared to the non-ATFGs. The red color represents positive functional connectivity. FWE corrected at *p* < 0.05.

The brain seed regions were selected based on the results obtained from the brain structure analysis. Thus, the AC training group showed no statistically significant changes compared to the other training groups. Compared to the non-ATFGs, the ATFGs had significantly increased FC between the TP and precuneus (Figure 5 and Table 4). Compared to the non-CTFGs, the CTFGs showed no statistically significant changes compared with the other training groups. In addition, we analyzed the relationships between behavior changes and brain structural changes. However, any changes of auditory and cognitive measures did not significantly correlate with changes of brain functional connectivity changes.

**TABLE 4:** The peak MNI coordinates and intensity of brain clusters with significance in brain connectivity.

Network and seed region	Brain region	Cluster size (mm^3^)	*p*-value FWE *p* < 0.05	Peak MNI coordinates (X, Y, Z)			Direction of correlation
**The main effect of the auditory factor**							
L. TP	Precuneus	184	0.019892	+ 12	–52	+ 50	Positive

*L. TP, Left temporal pole; AC, auditory-cognitive training; A, auditory training; C, cognitive training. FWE corrected at p < 0.05.*

## Discussion

In this study, we investigated the beneficial effects of AC training on cognitive functions (via LM, D-CAT, and DS), auditory performance (via PTA), and MRI measures (brain structure and FC) in healthy older adults. We found three main results related to our hypotheses. First, AC training led to a change in rGMV in the frontal regions but did not improve cognitive and auditory performance compared to the other groups. Second, the ATFGs improved auditory performance (PTA threshold), changed the rGMV in the L. TP, and increased the FC between the L. TP and the precuneus compared to the non-ATFGs. Third, the CTFGs improved cognitive performance in terms of LM and D-CAT and changed the rGMV in the R. DLPFC, L. ITG, OFC, and right cerebellum (lobule 7 Crus 1) compared to the non-CTFGs. We have discussed these findings separately below.

In the first finding, AC training showed neural plastic changes in the rGMV, but did not improve any cognitive function or auditory performance compared to the other training groups. This finding partially supports our hypothesis. For neural plastic changes, we found that AC training increased the rGMV in the R. DLPFC, L. ITG, OFC, and right cerebellum (lobule 7 Crus 1). The DLPFC is suggested to be involved in central executive processes (Hertrich et al., 2021). Particularly relevant to multiple tasks, the DLPFC is involved in scheduling processes in complex tasks (task management) (Smith and Jonides, 1999). Moreover, our results are consistent with previous findings in a multitasking cognitive training study (two or more cognitive activities at the same time); multitasking cognitive training using an auditory stimulus and a visual stimulus tasks increased the rGMV in the DLPFC in healthy young adults after a 4-week training period (Takeuchi et al., 2014). Additionally, previous neuroimaging studies have reported that the OFC, ITG, and cerebellum (lobule 7 Crus 1) are important for the integration of visual and auditory information (Wu et al., 2013; Nogueira et al., 2017; Lin et al., 2020). The OFC is thought to play an important role in adaptation to goal-directed behavior (Furuyashiki and Gallagher, 2007). During AC training, the participants had to integrate multisensory information during the combination of auditory and cognitive training factors. Therefore, the rGMV in the R. DLPFC, L. ITG, OFC, and right cerebellum (lobule 7 Crus 1) increased after 4 weeks of AC training. We did not find any significant beneficial effects of AC training on cognitive function and auditory performance compared to other training groups. This result is inconsistent with previous findings (Yusof et al., 2019). The training duration might be one of the reasons for this inconsistency, as the previous study had an 8-week intervention period, compared with the 4 weeks in the present study.

In the second finding, the ATFGs improved auditory performance (PTA threshold), changed rGMV-related speech perception, and increased brain connectivity in regions related to listening effort and language processing compared to the non-ATFGs. These findings support the second hypothesis of the present study. It was been previously reported that older adults could discriminate between words and sentences in high-noise situations after a 4-week training period (Karawani et al., 2016). However, the current study is the first to report that older adults can also listen to a sound with low intensity level, as shown with the PTA threshold.

The ATFG brain imaging results showed an increase in the rGMV in the left TP compared to that of the non-ATFGs. A previous fMRI study used sentence-listening tasks for normal hearing and listening difficulty (Stewart et al., 2020); it included three contrasts (phonology, intelligibility, and semantics). The phonology contrast showed bilateral activation in the middle and superior temporal gyrus, including the Heschl’s gyrus and TP. Phonology is a system of processing the smallest units of speech sounds and their linguistic combinations. As per previous findings (Khalfa et al., 2001), the TP descending influence may improve the auditory afferent message by adapting the hearing function according to the cortical analysis of the ascending input. Previous studies, as well as the current study, have shown that listening to adverse conditions increases the activity of the anterior temporal cortex regions, specifically in the TP. In the present study, the participants in the ATFGs required auditory effort because the sound intensity level decreased during the auditory training task. In terms of FC, the ATFGs showed a significant increase in FC between the L. TP and precuneus compared to the non-ATFGs. The FC between the TP and precuneus is reportedly important in hearing sounds in situations requiring high auditory effort (Rosemann and Thiel, 2019). A study has suggested that the FC between the TP and precuneus is associated with auditory effort (Rosemann and Thiel, 2019). In the ATFGs, the participants focused on low sound intensity level during the auditory training tasks. Therefore, FC between the TP and precuneus was comparable between the ATFGs and non-ATFGs.

In the third finding, the CTFGs improved cognitive performance and increased the rGMV in the R. ITG, R. IOG, and right cerebellum (lobule 7 Crus 1) compared to the non-CTFGs. These results support the third hypothesis in present study. For cognitive improvements, the CTFGs showed significant improvements in the LM and D-CAT. Multiple cognitive training usually presents better results than single cognitive training (Auffray and Juhel, 2001). In addition, previous studies that used cognitive training reported near transfers in cognitive function (Golino et al., 2017). In this study, our cognitive training included several cognitive components, such as attention, episodic memory, and working memory. Therefore, we found significant improvements in episodic memory and attention performance.

The CTFGs showed a significant increase in the rGMV in the R. ITG, R. IOG, and right cerebellum (lobule 7 Crus 1) compared to the non-CTFGs. Previous functional neuroimaging studies have shown that the L. ITG should be recruited more for the maintenance of words than pseudowords (Fiebach et al., 2006). In studies of language processing, the ITG has also been associated with prelexical processing of abstract word form (Cohen et al., 2000) and conceptual semantic processing (Herbster et al., 1997), independent of presentation modality (Cohen et al., 2004). The previous training study supports the suggestion that the cerebellum may be important for shifting performance from the attentionally demanding stage to a more automatic state (Holtzer et al., 2017). Previous neuroimaging studies have reported that the ITG and cerebellum (lobule 7 Crus 1) are associated with information integration (Wu et al., 2013; Nogueira et al., 2017; Lin et al., 2020). Additionally, a previous study reported that activity occurs in the IOG during tasks that require episodic memory usage (Matthaus et al., 2012). The participants in the CTFGs were required to exert more cognitive effort as the cognitive training tasks increased in difficulty. Therefore, brain imaging results showed an increase in the rGMV in the R. ITG, R. IOG, and right cerebellum (lobule 7 Crus 1).

The present study had some limitations. First, AC training had fewer beneficial effects on behavioral performance compared to other training modalities. However, we found positive effects on the brain structure and FC. A possible explanation for this could be the different measurement indices used (cognitive, auditory, and brain) had an effect. Second, we did not consider the effects that could occur over time after training. Third, the present study did not evaluate the beneficial effects of training on quality of life. As mentioned previously, ARHL causes a cascade of deficits that can lead to dementia. Thus, after the present training, the participants may have affected communicating in quality of life and change in social isolation. It would be beneficial is a future study would consider the quality of life effects of AC training. Fourth, the PTA was used to assess auditory sensitivity, but the cognitive potential noted cannot be excluded. Fifth, the auditory and cognitive training factors increased the level of difficulty in the training groups relative to the subject‘s performance. However, the auditory training factor may partially lead to a change in the difficulty of the cognitive task, especially considering that the study group consisted of older adults. Sixth, for the working memory and short-term/episodic memory tasks, it is also necessary to check whether the same effect can be achieved by changing the stimulus of the training task. Final limitation is the small number of participants. Due to the small sample number of samples, it is hard to generalize the current results into general a population. This is a first step in the overall research on AC training using low sound intensity level as auditory training task. In the future study it is important to conduct a large sample RCT to investigate beneficial effects of AC training on auditory and cognitive performance as well as neural plasticity.

Hearing aids are the first choice for people with hearing loss and have made significant technological advances over the last two decades. Although satisfaction with hearing aids has improved, hearing aid users often encounter difficulties in challenging listening conditions (Ohlenforst et al., 2017). The disadvantages of hearing aids include the following: (1) they do not block background noise, (2) separate speech from sounds in noisy environments, and (3) they allow users to hear sounds at a distance (Ohlenforst et al., 2017). Thus, the current auditory training method have important implications for the clinical management of people with deterioration in auditory processing. The present study showed that the auditory training to increase auditory performance. Especially, AC training changed the brain structure in the DLPFC and ITG, which are associated with working memory and auditory processing. We believe that this study has implications for improving auditory performance in older adults. In addition, the present training methods may improve auditory sensitivity and alter brain structure and functional connectivity.

## Data Availability Statement

The raw data supporting the conclusions of this article will be made available by the authors, without undue reservation.

## Ethics Statement

The studies involving human participants were reviewed and approved by the Institutional Review Board at the Tohoku University of Sendai, Japan. The participants provided their written informed consent to participate in this study.

## Author Contributions

NYSK: conceptualization, data curation, formal analysis, investigation, visualization, writing—original draft, and writing—review and editing. RN: conceptualization, investigation, methodology, validation, visualization, writing—original draft, and writing—review and editing. KO and YM: investigation, writing—review and editing. RK: supervision and writing—review and editing. All authors contributed to the article and approved the submitted version.

## Conflict of Interest

The authors declare that the research was conducted in the absence of any commercial or financial relationships that could be construed as a potential conflict of interest.

## Publisher’s Note

All claims expressed in this article are solely those of the authors and do not necessarily represent those of their affiliated organizations, or those of the publisher, the editors and the reviewers. Any product that may be evaluated in this article, or claim that may be made by its manufacturer, is not guaranteed or endorsed by the publisher.

## Footnotes

1
https://www.graphpad.com/quickcalcs/

2
http://www.neuro.uni-jena.de/cat/

3
http://dbm.neuro.uni-jena.de/tfce/

4
https://www.nitrc.org/projects/artifact_detect
