# Supplementary material for: Auditory Cognitive Training Improves Brain Plasticity in Healthy Older Adults: Evidence From a Randomized Controlled Trial
Source: Front Aging Neurosci. 2022 Mar 31;14:826672. doi: 10.3389/fnagi.2022.826672 (PMC9010026; doi:10.3389/fnagi.2022.826672)

Supplementary Material

# Auditory Measures

*Pure-tone audiometry (PTA):* PTA measurement were conducted for conventional audiometric frequencies of 125 Hz, 250 Hz, 500 Hz, 1 kHz, 2 kHz, 4 kHz, and 8 kHz. According to the Japanese standards, measurements at 3 kHz and 6 kHz were not included. The order of the participants’ assessment started with the right ear. The data for the better ear were included as outcome measures in the analysis. The Japan Audiological Society used different audiological criteria for normal hearing (< 25 dB), mild (25 – 39 dB), moderate (40 – 69 dB), severe (70 – 89 dB), and profound (≥90 dB).

*Speech-recognition threshold (SRT):*SRT is the lowest hearing level at which 50% of the words presented can be identified correctly. The goal is to find the softest sound level at which one can hear and repeat approximately one-half of the compound words correctly. The guidelines of the Japan Audiological Society used single-digit numbers (2 “ni”, 3 “san”, 4 “yon”, 5 “go”, 6 “roku”, and 7 “nana”). It was created and used to measure the threshold for listening to speech-by-speech (speech understanding threshold). The participants were asked to write a single-digit number presented to their ears.

# Cognitive Function Measures

*Mini-Mental State Examination (MMSE):* The MMSE (Folstein et al., 1975) is a widely used cognitive function test among older adults. MMSE scores suggest a decline in global cognitive function. It contains tests of orientation, attention, memory, language, and visual-spatial skills. The MMSE is a 20-item instrument and the test score is from 0 to 30. Lower scores (<26) indicate the degrees of general cognitive dysfunction. The primary measure was the total score of this assessment (max = 30).

*Logical memory (LM):* “LM evaluates the performance of episodic memory. LM is a subtest of the Wechsler Memory Scale-Revised (WMS-R) (Wechsler, 1987). LM consists of two short-paragraph stories (Story A and Story B). In LM, participants must memorize short stories. The stories were scored in terms of the number of story units recalled, as specified in the WMS-R scoring protocol. We used either Story A or Story B. The primary measure for this task was the number of correct story units recalled.“ (Nouchi, Taki, et al., 2012a).

*Digit span (DS):* “The DS is a subtest in Wechsler Adult Intelligence Scale-Third Edition (WAIS-III). DS measures working memory by requiring participants to memorize numbers and repeat the numbers in inverse order. DS-F has 16 sequences. This test’s primary measures are raw scores that reflect the number of correctly repeated sequences until the discontinue criterion (i.e., failure to reproduce two sequences of equal length) is met (Wechsler, 1997). The maximum raw score of DS-F is 16.” (Nouchi, Taki, et al., 2012a).

*Digit cancellation test (D-CAT):* The D-CAT evaluates attention (Hatta T, 2000). The test sheet consisted of 12 rows of 50 digits. Each row contains five sets of numbers from 0 to 9 arranged in random order. Consequently, any digit appears five times in each row with randomly determined neighbors. The D-CAT consists of three such sheets. Participants are instructed to search for the target number (s) that had been specified to them and to delete each one with a slash mark as quickly and as accurately as possible until the experimenter sends a stop signal. These three trials, first with a single target number (6), second with two target numbers (9 and 4), and third with three (8, 3, and 7). Each trial is performed for 1 min. Consequently, the total time required for the D-CAT is 3 min. In the second and third trials, it is emphasized that all the target numbers instructed should be canceled without omission. The primary measure of this test is the number of hits (correct answers). We use only the number of hits in the first trial.

# Training Materials

*Task stimuli:* We recorded the voices of two female and two male Japanese speakers. The voices were recorded while they read a worklist from an A4 format sheet. The recording consisted of numbers (e.g. “1” /ichi/, “2” /ni/, “3” /san/, “4” /yon/, “5” /go/, “6” /roku/, “7” /nana/, “8” /hachi/, and “9” /kyu/), polysyllabic words with high-frequency (e.g. “table” /tsukue/), and vowels (e.g. /a/, /e/, /i/, /o/, and /u/). They were invited to maintain a flat tone of voice and pronounce the items as clearly as possible. If the experimenter judged that the recorded items were not easily discriminable, they then asked the speakers to repeat them until they were. The voice was recorded in “wav” file format using Audacity software (<https://www.audacityteam.org/>) at the rate of 44.100 samples per second with a Panasonic laptop computer (CF-RZ). Sixteen bits were allocated to each sample. Voice samples obtained from each speaker were recorded in a soundproof room and using an omnidirectional AT2020USB+PK microphone (Audio-Technica, Tokyo, Japan). The microphone was positioned approximately 2.5 cm from the speaker’s mouth, maintaining a microphone-to-mouth angle of approximately 90°. The mean duration of each stimulus was 2-8 ms. Each number and word was pronounced slowly (normal speed). We used the word list from the previous study by NTT Basic Research Laboratories (Amano, Kondo, & Kakehi, 1995). All audio stimuli were presented to both slides (two ears) at once via headphones (BOSE QC 35 II).

The training sessions were performed as three tasks (short-term memory, working memory, and attention task). All training tasks were controlled using E-Prime 3.0 software and collected information on oral responses, button presses, and reaction times (RTs). When starting the training program, instructions regarding the task were shown on a screen (white letters on black background). The trial started when the participant agreed and pressed the button to proceed. Each trial started with a central fixation point (5 ms duration), followed by a presentation of the audio stimuli (audio stimuli varied depending on each task’s difficulty level). After the audio stimuli were presented, one screen of answer instruction was presented (for working memory task: “Repeat what you heard in inverse order. When finished, press the red button to proceed.”; for the short-term memory task: “Repeat what you’ve heard in the same order. When finished, press the red button to proceed.”). The subsequent trial started only after the participants pressed the red button. In the working memory task and short-term memory task, the participants’ oral responses were recorded. After the instruction screen, a central fixation point was presented in the attention task while the audio stimulus was played. All participants were instructed to press the button when listening to the audio stimulus, and they were instructed to respond as accurately and as quickly as possible. The participants used their right hand to press the button.

On the training day, it was possible to train two participants simultaneously for one hour (two soundproof rooms were made available; Room A and Room B). The second participant started training with a delay of 5 minutes. The 5 minutes delay was computed in the setup. Before entering the soundproof room, the examiner asked the participant to tour off their cell phone if they had one. The participant sat in front of the PC screen at a distance of approximately 60 cm, and the microphone was positioned approximately 20 cm from the participant’s mouth. He/she was required to press a previously assigned response key button with the right hand or to respond orally when necessary. The correct placement of the headphones was confirmed each time by the examiner. Between each sub-session training, there was a 5 minutes break. Each sub-session consisted of a program with three tasks.

*Experimental setup:* The training session was carried out in two soundproof rooms (YAMAHA Corporation) positioned side by side (Room A and Room B). At each session, the acoustic treatment soundproof room was occupied by one participant. The internal dimensions of the soundproof room were 1.766 mm x 2.648 mm. Within each soundproof room, a table and chair were positioned in the center. To maintain air circulation inside the room, a portable fan was placed. The table had a 40-inch display, a keyboard, a mouse, headphones (BOSE QC 35 II), a microphone, and an audio volume control system. To adjust the audio volume every 0.5 dB, the Grace Design m905 system (Grace Design Corporation) was used. The trainer controlled the session in the soundproof room from the outside. Therefore, all USB cables (keyboard, mouse, microphone, and audio input volume controller) were connected to a 4-port adapter with a 5 m-extension. The monitor HDMI cable was connected similarly. The extension cables passed through an opening of the soundproof room and connected to a laptop (MOUSE B505H-S1). Outside the soundproof room, a trained examiner was responsible for guiding each participant’s training sessions. Regarding the equipment’s maintenance and hygiene after each participant’s training session, the examiner sterilized the keyboard, mouse, microphone, chair, and table with alcohol. A metal plate was placed in front of the microphone to comply with hygiene requirements.

**4 Analyses**

In addition, we performed one-sample t-tests with permutation to investigate whether the change scores in each group was a significant different from zero. We found the following results (auditory-cognitive training group (LM [p = 0.673]; DS [p = 0.0428]; D-CAT [p = 0.0728]; PTA [p = 0.24]), auditory training group (LM [p = 0.0166]; DS [p = 0.547]; D-CAT [p = 0.587]; PTA [p = 0.0596]), cognitive training group (LM [p = 0.766]; DS [p = 0.14]; D-CAT [p = 0.363]; PTA [p = 0.0548]), and active control training group (LM [p = 0.0176]; DS [p = 0.454]; D-CAT [p = 0.143]; PTA [p= 0.315]), auditory training factor (LM [p = 0.589]; DS [p = 0.058]; D-CAT [p = 0.0744]; PTA [p = 0.0134]), and cognitive training factor (LM [p = 0.0408]; DS [p = 0.0386]; D-CAT [p = 0.0948]; PTA [p = 0.013])).

**5. Figures**

**Histograms of the change scores of study measurements (logical memory test, LM; digit cancellation test, D-CAT; digit span, DS; pure-tone audiometry, PTA).**


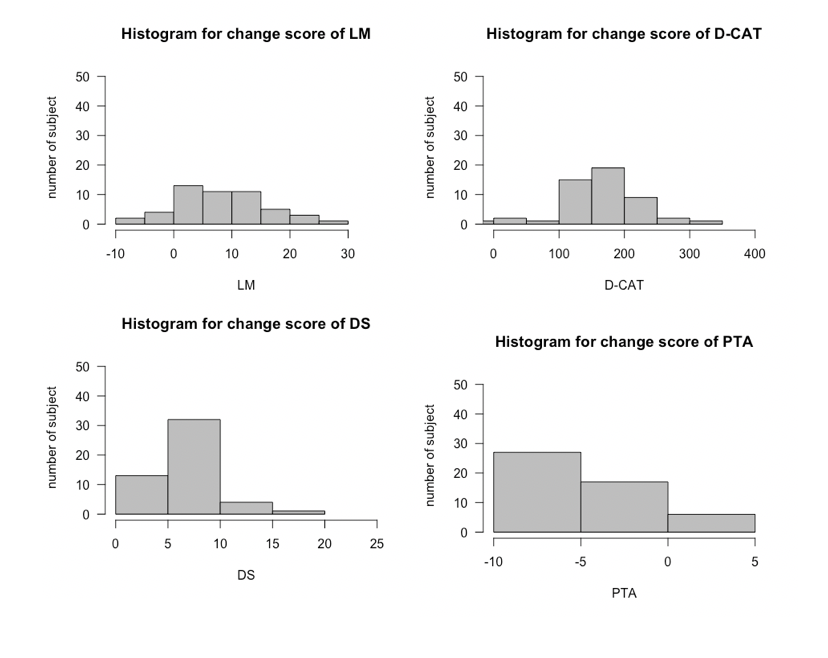

Supplement: Supplementary file 1 [file Data_Sheet_1.docx]
